# Evaluation of the biotechnological potential of peptide Cupiennin 1a and analogs

**DOI:** 10.3389/fmicb.2022.850007

**Published:** 2022-08-18

**Authors:** Rayssa Oliveira Araújo, Michel Lopes Leite, Thais Tavares Baraviera Dutra, Nicolau Brito da Cunha, Taia Maria Berto Rezende, Marcelo Henrique Soller Ramada, Simoni Campos Dias

**Affiliations:** ^1^Centro de Análises Proteômicas e Bioquímicas, Pós-Graduação em Ciências Genômicas e Biotecnologia, Universidade Católica de Brasília, Brasília, Brazil; ^2^Faculdade de Agronomia e Medicina Veterinária, Universidade de Brasília - UnB, Brasília, Brazil; ^3^Pós-Graduação em Ciências da Saúde, Universidade de Brasília, Brasília, Brazil; ^4^Programa de Pós-Graduação em Gerontologia, Universidade Católica de Brasília, Brasília, Brazil; ^5^Pós-Graduação ao em Biologia Animal, Campus Universitário Darcy Ribeiro, Universidade de Brasília, Brasília, Brazil

**Keywords:** antimicrobial peptides, rational design, analogs, spider, cytotoxicity

## Abstract

Antimicrobial peptides (AMPs) are components in the innate immune system of various organisms, and many AMPs can be found in poisons from animals such as spiders, scorpions, and snakes. The peptide Cupiennin-1a is present in the venom of the spider *Cupiennius salei* and belongs to a group of peptides called cupiennins. The peptide demonstrated high cytotoxic activity against mammalian cells; thus, aiming to solve this problem, seven analogs were designed (R1a, R1b, R2b, R3b, R6b, R8b, and R10b) based on the primary structure of the peptide Cupiennin 1a, reducing its size and substituting some amino acid residues. The antimicrobial results showed that all Cupiennin 1a analogs displayed antimicrobial activity against the tested bacterial and fungal strains. Cytotoxicity tests demonstrated a decrease in the cytotoxic effect of the analogs when compared to the peptide Cupiennin-1a. The antitumor activity against breast adenocarcinoma lines was observed for all the peptides, displaying a better effect against the MCF-7 and MDAMB-231 cell lines. The eight peptides have insecticidal potential, and the original peptide and analogs R6b, R8b, and R10b showed better efficiency even at low concentrations. The rational design of the analogs led to new molecules displaying activities against different cell types and reduced cytotoxicity toward healthy mammalian cells when compared to the original peptide, demonstrating that this was an interesting approach for the development of molecules with biotechnological potential.

## Introduction

Due to the continued emergence of microorganisms that are increasingly resistant to current antibiotics, numerous concerns about existing therapeutic agents for the treatment of autoimmune diseases, and widespread problems with pests in agriculture, many antimicrobial molecules have been studied worldwide. In this context, antimicrobial peptides appear as a new biotechnological tool for therapeutic use because these molecules can be selective, efficient, and fast-acting (Le, 2017).

Antimicrobial peptides (AMPs) are molecules present in the innate immune system of various organisms. These molecules have a considerable variation in the composition of their amino acid residues, forming different secondary structures, contributing to the diverse biological activities they can display. AMPs are low molecular weight molecules with a wide spectrum of activities, and may inhibit bacteria, viruses, fungi, tumor cells, and protozoa (Zhao et al., 2013) and can act as immunomodulators (Boparai and Sharma, 2019). Most AMPs have amphiphilicity characteristics that allow them to be soluble in aqueous environments (Adem Bahar, 2015).

Among the most abundant AMPs in nature, those with cationic alpha-helical structures stand out, as these can disturb the cytoplasmic membrane by osmotic shock, leading to cell lysis. The main AMPs known for this capability are cecropin, magainin, human cathelicidin LL-37, and its derivatives, in addition to proline-rich antimicrobial peptides (prAMPs; Graf et al., 2017; Le, 2017). Physicochemical characteristics and secondary structure contribute to a good selectivity and efficacy on cytoplasmic membranes, making these molecules a target for the generation of new biological agents (Torres et al., 2019).

Among the various animal-derived AMPs with biotechnological potential, we highlight the peptide Cupiennin-1a, also called M-ctenitoxin-Cs1a or Cu-1a, present in the venom of the wandering spider *C. salei*. Described by Kuhn-Nentwig et al. (2002), the peptide presents 35 amino acid residues, with a mostly hydrophobic N-terminal region and a polar/charged C-terminal. It is a cationic peptide with a global charge of +8, an amidated C-terminal, presenting a high potential helix formation in the presence of trifluoroethanol (TFE; Kuhn-Nentwig et al., 2002).

Peptides of the cupiennin group act by cytolytic mechanisms against a broad spectrum of bacteria, such as *Staphylococcus aureus, Pseudomonas aeruginosa, Escherichia coli, Enterococcus faecalis* (Kuhn-Nentwig et al., 2002), *S. epidermidis, Bacillus subtilis, P. putida*, and *Paracoccus denitrificans* (Haeberli et al., 2000). Thus, it was suggested that the role of cupiennin in the venom of *C. salei* could be to protect the poison apparatus (glands and ducts) against infections, as well as to enhance neurotoxin interaction with intracellular targets (Kuhn-Nentwig et al., 2015). However, Cupiennin-1a (Cu-1a) has a wide range of other biological activities, such as its insecticidal action against flies of the species *Drosophila melanogaster* (Kuhn-Nentwig et al., 2002), and parasitic activity against *Trypanosoma cruzi, Trypanosoma brucei*, and *Plasmodium falciparum* (Kuhn-Nentwig et al., 2013).

This peptide shows high cytotoxicity to human erythrocytes, which makes it difficult to use as a therapeutic agent. Thus, the rational design of potential antimicrobial peptides would be an attempt to optimize toxicity for therapeutic use and the search for new biotechnological activities (Porto et al., 2012). This work aimed to select the Cu-1a peptide sequence as a template for the rational design of 7 molecules, in order to reduce the toxicity of the original peptide without losing the biotechnological potential already described in the literature.

## Materials and methods

### Synthesis of Cu-1a peptide and its analogs

The antimicrobial peptide Cu-1a was synthesized according to the amino acid sequence GFGALFKFLAKKVAKTVAKQAAKQGAKYVVNKQME-NH2 (where NH2 indicates an amidated C-terminal) of the peptide isolated from *C. salei* spider venom (Kuhn-Nentwig et al., 2002) by Aminotech Company (Campinas, São Paulo—Brazil). The synthesis was carried out using solid-phase approach and F-Moc methodology (Maggiora et al., 1992). Then, seven analogs were designed based on the deletion and substitution of amino acid residues from the original Cu-1a peptide sequence to maintain the desired characteristics for analogous molecules and subsequently synthesized as previously described for Cu-1a.

### Molecular mass analysis, quantification, and helix projection

The masses of the 8 peptides were analyzed by mass spectrometry using a MALDI-TOF/TOF (Matrix Assisted Laser Desorption Ionization—Time of Flight) Autoflex Speed (Bruker Daltonics). Peptides were diluted in ultrapure water and mixed with a solution of α-cyano-4-hydroxycinnamic acid matrix at 10 mg.ml^−1^ (50% (v/v) acetonitrile, 0.3% (v/v) trifluoroacetic acid) in a 1:3 ratio. Subsequently, this mixture was deposited in triplicate on an MTP384 ground steel plate and maintained at room temperature until its total crystallization. The mass/charge ratio (m/z) of the peptides was obtained in the range of 700–5,600 m/z, in reflected, positive mode, after external calibration of the equipment with Peptide Calibration Standard II (Bruker Daltonics). MS/MS spectra were obtained *via* LIFT fragmentation (Suckau et al., 2003), and *de novo* interpretation for amino acid sequence confirmation was performed using FlexAnalysis software (Bruker Daltonics).

To perform the experiments, each peptide was diluted in Milli-Q water and quantified by UV absorption at 205, 215, and 225 nm, using the concentration formula (Murphy and Kies, 1960).

A = (225 – 215) × 0.144.

B = 205 × 0.31.

A + B/2 = [] mg.ml^−1^.

After quantification, peptides were aliquoted at a concentration of 128 μg.ml^−1^.

The projection of the helical wheel of peptides was performed using NetWheels,^1^ developed by the Institute of Biology at the University of Brasília.

### Evaluation of antimicrobial activity against bacteria

Susceptibility tests were performed using clinical isolates *of Klebsiella pneumoniae carbapenemase* (KPC 1410503) and methicillin-resistant *S. aureus* (MRSA 3730592). Tests were also performed with ATCC (American Type Culture Collection) isolates of *K. pneumoniae* (ATCC 13883) and *S. aureus* (ATCC 25923).

Two multidrug-resistant clinical isolates and two ATCC isolates were chosen to perform microbial susceptibility testing as models for studies with bacteria susceptible to antimicrobials found in the Gram-Positive and Gram-Negative groups of bacteria.

The broth microdilution method was performed according to the Clinical and Laboratory Standards Institute (CLSI) M07-A10 protocol to determine the Minimum Inhibitory Concentration (MIC; Clinical Laboratory Standards Institute, 2015). The assay was performed on 96-well microplates (TPP, United States), containing 5×10^5^ CFU.ml^−1^ of each tested bacterium per well and different peptide micromolar concentrations (Table 1) in a final volume of 100 μl of Mueller-Hinton Broth (MH). Negative control was represented by bacteria incubated in MH medium, while positive control was represented by culture incubated in MH medium with Amikacin. Plates were kept under agitation at 37°C in a BioTek spectrophotometer (PowerWaveTM HT Microplate Reader) with optical density (OD) readings at 595 nm every 30 min for 24 h. Inhibition results were analyzed using GraphPad Prism 8 software by comparing the OD from negative control wells (100%) to those containing peptides. All tests were performed in biological triplicates.

**Table 1:** Concentrations in μM and μg.ml^−1^ used in activity assays against bacteria, fungi, RAW264.7 cells, and tumor cells.

Peptides	Concentration μM	Concentration μg.ml^−1^^*^
Cu-1a	33–4.22	128–16
R1a	63.26–7.90	128–16
R1b	67.54–8.44	128–16
R2b	68.62–8.57	128–16
R3b	67.21–8.40	128–16
R6b	70.8–8.85	128–16
R8b	70.43–8.80	128–16
R10b	63.3–8.53	128–16

* These concentrations refer to micromolar ratios between 128 and 16 μg.ml^−1^.

### Evaluation of antimicrobial activity against *Candida parapsilosis*

The broth microdilution method was performed according to the Clinical and Laboratory Standards Institute Guideline M27-A3 with modifications to determine the Minimum Inhibitory Concentration (MIC; Clinical Laboratory Standards Institute, 2008).

Initially, the fungus *C. parapsilosis* (ATCC22019) was grown in a Petri dish containing Sabouraud Dextrose Agar medium (Dextrose 40 g/L; Peptic digestion of animal tissue 5.0 g.L^−1^; and Agar 15 g.L^−1^) for 48 h. Subsequently, a pre-inoculum was performed from a yeast colony that was placed in 10 ml of liquid Sabouraud medium and incubated for 16 h at 28°C, with a constant rotation of 200 rpm. After the incubation period, 2.5 × 10^3^ CFU.ml^−1^ of fungus per well was incubated with different peptide micromolar concentrations (Table 1) to assess the MIC for each peptide.

Fungal cells incubated with Sabouraud medium were used as negative control, while positive control was represented by fungal cells incubated in Sabouraud medium and amphotericin B (10 μM). Subsequently, plates were incubated for 24 h at 30°C. After this period, the absorbance was read (595 nm) in a BioTek spectrophotometer (PowerWaveTM HT Microplate Reader), and data of antifungal activity with percentage of inhibition of peptides were plotted in GraphPad Prism 8 Software.

### RAW 264.7 and human fibroblast cell line culture

The cell line RAW 264.7 was obtained from the Rio de Janeiro Cell Bank (CR108). These cells are macrophages derived from induced tumors in male BALB/c mice infected with Abelson’s murine leukemia virus (Raschke et al., 1978). RAW264.7 cells were grown in DMEM medium (Dulbecco’s Modified Eagle’s medium, composed of CaCl_2_ (anhydrous) 200 mg.ml^−1^, Fe(NO_3_).9H_2_O 0.1 mg.ml^−1^, KCL 400 mg.ml^−1^, MgSO_4_ (anhydrous) 97.67 mg.ml^−1^, NaCL 6.400 mg.ml^−1^, NaH_2_PO_4_.H_2_O 125 mg.ml^−1^) supplemented with 10% (v/v) of bovine fetal serum, 0.5% (v/v) of DMEM amino acid solution, 0.05% (w/v) gentamicin, 0.5% (w/v) of L-glutamine, and 0.5% (w/v) of penicillin/streptomycin (1,000 U.ml^−1^) in an incubator containing 5% CO_2_, at 37°C, and 95% humidity (Raschke et al., 1978; Nguyen et al., 2017).

Human fibroblast (Hfib) cell cultures were cultivated in Dulbecco’s Modified Eagle Medium (DMEM; Sigma-Aldrich, United States) supplemented with 10% fetal bovine serum (Sigma-Aldrich). Hfib were cultured in a humidified incubator containing 5% CO_2_ at 37°C and 95% humidity.

### Cytotoxicity tests against RAW 264.7 and Hfib

RAW 264.7 cells were seeded in 96-well plates, at a concentration of 10^4^ cells/well, and incubated for 24 h. Each peptide was subsequently added at different micromolar concentrations (Table 1). Positive control was performed by cells in culture medium and negative control, as cell culture in lysis solution (10 mM Tris, 1 mM EDTA, and 0.1% Triton X-100, pH 7.4).

Hfib cell line was seeded onto 96-well plates (1 × 10^3^ cells/well, 100 μl) before treatment and allowed to adhere and grow over the 24 h period. Each peptide was subsequently added at different micromolar concentrations. The results were evaluated using GraphPad Prism 8 software, where a One-Way ANOVA statistical test followed by Bonferroni’s multiple comparisons test was performed.

Cell viability analyses were performed in technical and biological replicates, and readings were obtained after 24 h of contact with peptides. At the end of the incubation period, MTT (Sigma-Aldrich, United States) was used to assess the ability of living cells to reduce salt 3-[4,5-dimethylthiazole-2-yl]-2,5-diphenyltetrazolium bromide for formazan production. Plates were read at 570 ηm (Mosmann, 1983) and results were expressed in the percentage of living cells. The results were analyzed using GraphPad Prism 8 software where a One-Way ANOVA statistical test was followed by Bonferroni’s multiple comparisons test.

### Nitric oxide production

After 24 h in the presence of peptides, RAW 264.7 cell supernatants were collected for nitric oxide (NO) evaluation. NO production was assessed by nitrite detection by Griess reaction (Mosmann, 1983), with modifications. One hundred microliters of cell culture supernatant were transferred to 96-well plates. A standard curve of nitrite was performed (200 – 0.097 mM). Then, 100 μl of a 1% (w/v) sulfanilamide solution was added in phosphoric acid 2.5% (w/v) and naphthylethylenediamine 1% (w/v) in phosphoric acid 2.5% (w/v), in the proportion of 1:1 to all wells. After 10 min of incubation at room temperature, plates were read at 490 ηm.

### Cytotoxicity tests against cancer cell lines

MDA-MB-231 and MCF-7 cell lines, both mammary adenocarcinomas, were cultivated in Roswell Park Memorial Institute (RPMI) 1,640 medium (Sigma-Aldrich, United States) supplemented with 10% fetal bovine serum (Sigma-Aldrich). Cell lines were cultured in a humidified incubator containing 5% CO_2_ at 37°C. Antitumor tests were performed on technical replicates and were assessed by MTT (Sigma-Aldrich, United States) assay, as described previously. Both cell lines were seeded onto 96-well plates (1 × 10^3^ cells/well, 100 μl) before treatment and allowed to adhere and grow over the 24 h period. Each peptide was subsequently added at different micromolar concentrations (Table 1). The results were evaluated using GraphPad Prism 8 software, where a One-Way ANOVA statistical test followed by Bonferroni’s multiple comparisons test was performed.

### Analysis of insecticidal activity

To evaluate the ability of the peptides to cause lysis and morphological changes in insect cells (Dutra, 2019; Patent: BR 102022 006138 6) as a first screening of the insecticidal activity of the peptides, in this work, the Spodoptera IPLB-SF-21AE _LVI-Cenargen cell line (Vaughn et al., 1977) was used and different concentrations of the peptides were added to the culture medium.

Cells were maintained at 27°C in TNMFH medium (Grace’s insect medium supplemented with lactalbumin hydrolyzate and yeastolate), supplemented with 10% (v/v) fetal bovine serum (Sigma-Aldrich; Castro et al., 2006).

Cell cultivation was carried out in 10 ml culture bottles, model K11-1050 25cm^2^, with 5 ml of culture medium per bottle. For the assays, the SF21 cells were grown in TNMFH medium, added 10% (v/v) of bovine fetal serum, and distributed in plates of 96 wells at the concentration of 2×10^4^ cells per well, after counting was performed in the Neubauer chamber. Cell suspension was distributed in a volume of 100 μl per well. Peptides were diluted in TNMFH medium and 100 μl of medium with peptide was added, obtaining concentrations of 10, 5, and 1 μM, reaching the volume of 200 μl at the final concentrations of reaction per well. Cells were placed in incubation at 27°C, for 24 h.

After 24 h, the cells were analyzed for their modifications, morphological alterations, turbidity of the medium, formation of clumps, and/or lysis in the presence of peptides, by observation under optical microscopy (Nikon Eclipse TS100) with 20× and 40× magnification. Characteristics that could represent cell death or cell dysfunction caused by the peptides were observed compared to control cells. Cells were evaluated by cell viability using the MTT test 3-[4,5-dimethylthiazole-2-yl]-2,5-diphenyltetrazolium bromide 0.5 mg.ml^−1^ (Sigma-Aldrich). To perform the test, the cell culture medium was discarded, and we added 100 μl of new medium. Ten μl of MTT was added to each well and incubated for 4 h, with the plates protected from light. After 4 h, we added 100 μl of DMSO to each well to dissolve the formazan crystals, and the reading in the microplate reader (Riss et al., 2016) was then performed. The results were analyzed using GraphPad Prism 8 software, where a One-Way ANOVA statistical test was followed by Bonferroni’s multiple comparisons test.

## Results

### Molecular mass analysis and helical wheel peptide projection

The seven analogs were generated excluding part of the C-terminal of the Cu-1a peptide described by Kuhn-Nentwig et al. (2002), producing molecules containing 18 or 19 amino acid residues, amino acid substitution was also performed along the peptide chain. The molecular mass and sequences of the seven synthesized analogs were analyzed by mass spectrometry using MALDI-TOF/TOF, Table 2 shows the results obtained.

**Table 2:** Sequence, number of amino acid residues, monoisotopic molecular weight [M + H+], charge, hydrophobicity (*H*), hydrophobic moment (μ*H*), and isoelectric point (pI) of the seven analog peptides.

Peptide	Sequence	Number of amino acid residues	[M + H]^+^	Charge	*H*	μ*H*	pI
^*^Cu-1a	GFGALFKFLAKKVAKTVAKQAAKQGAKYVVNKQME-NH _2_	35	3798.63	8	−0.138	0.0226	11.30
R1a	GFGALFKFLAKKVAKTVA**K**-NH_2_	19	2023.34	5	−0.2579	3.1033	11.1
R1b	GFGALFKFLAKKVAKTVA-NH_2_	18	1895.14	4	0.2778	3.1243	11
R2b	GFGALFKFLAKKVAK**A**VA-NH_2_	18	1865.13	4	0.4278	3.2387	11
R3b	GFGALFK**R**LAKKVAKTVA-NH_2_	18	1904.32	5	−0.8333	3.8198	11.8
R6b	GFGALFKFLAK**A**VAK**A**VA-NH_2_	18	1807.98	3	0.9167	3.3629	10.8
R8b	GFGALFK**R**LAK**A**VAK**A**VA-NH_2_	18	1817.21	4	−0.1944	3.9049	11.8
R10b	GFG**K**LFK**R**LAK**A**VAK**A**VA-NH_2_	18	1874.13	5	−0.6833	4.3819	11.8

* Data reported by Kuhn-Nentwig et al. (2002), Hydrophobicity (*H*) and Hydrophobic Moment (μ*H*) data were described by the total mean values of each characteristic by the authors.Modifications to amino acid residues made in the primary peptide sequence are marked in blue (Ala) and red (Arg) and green (Lys) along the generated sequence.

Physicochemical characteristics such as cationicity, helicity, hydrophobicity, hydrophobic moment, extension of the peptide chain, and c-terminal amides were taken into account for the rational design of these analogs. The analogs generated are positively charged peptides as well as the Cu-1a peptide. Arginine (R), Lysine (K), and Alanine (A) were the amino acids chosen for substitution along the peptide chain due to their potential to contribute to the formation of idealized amphipathic helixes. These amino acids can also contribute to the bending of peptides, because the interactions between the lateral chains can stabilize helical structures (Deslouches et al., 2013).

Finally, the analogs also presented an amidated C-terminal, as well as the original molecule. There are several ways to visualize structural conformations adopted by peptides, two of which are commonly used by biochemists for two-dimensional visualization of secondary molecule structures such as helical wheels and Wenxiang diagrams (Wadhwa et al., 2018). In this work, we chose two-dimensional visualization using helical wheels with software for the projection of the helical wheel, such as NetWheels.^2^

**Figure 1:**
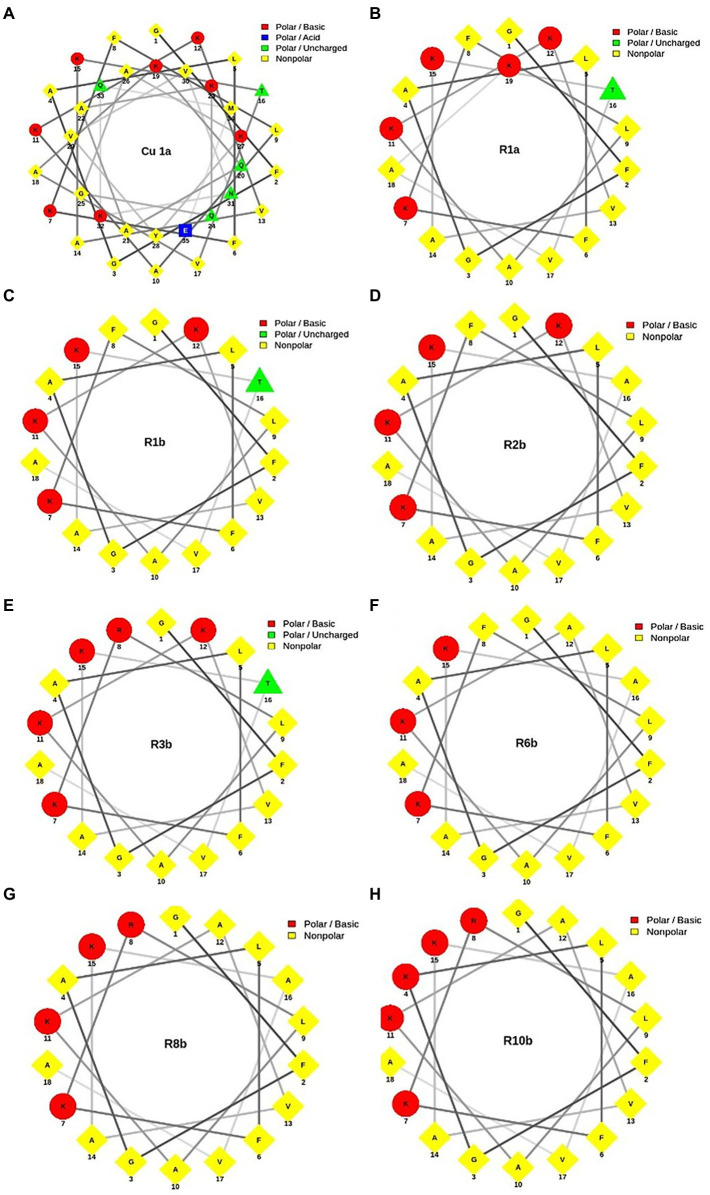
Helical wheel projection of analogous synthetic peptides designed based on the Cu-1a peptide sequence. In red, the basic polar amino acid residues; in blue, the acid polar amino acids; in green, the uncharged polar amino acids; and in yellow, the nonpolar amino acids. **(A)** Cu-1a; **(B)** R1a; **(C)** R1b; **(D)** R2b; **(E)** R3b; **(F)** R6b; **(G)** R8b; and **(H)** R10b.

Figure 1 shows the helical projection of the wheel of the eight synthetic peptides, highlighting in red the basic residues of polar amino acids, in blue, the acidic polar amino acids, in green, the unloaded polar amino acids, and, finally, in yellow, the hydrophobic amino acids. It is also possible to observe that the hydrophilic (red) and hydrophobic (yellow) part that make up each peptide are well distributed. Through these regions, we see that the generated molecules have amphipathic characteristics that can contribute to the biological activity of peptides.

### Antimicrobial activity against bacteria and fungi

Antimicrobial assays showed that Cu-1a and its analogs showed activity against bacteria and fungi at different concentrations.

Cu-1a and analogs R1a, R2b, R6b, R8b, and R10b showed the best antimicrobial results, and analog R10b was the most efficient of them against both strains of *S. aureus*. Except for the Cu-1a peptide, there was no change in the concentration required to inhibit the growth of *S. aureus* ATCC25923 and MRSA strain by analogs (Table 3).

**Table 3:** MIC values found for peptide Cu 1a and their analogs against bacteria and fungi.

Bacterial strain	Minimum inhibitory concentration (MIC) (μM)							
	Cu-1a	R1a	R1b	R2b	R3b	R6b	R8b	R10b
*K. pneumoniae carbapenemase—KPC*	16.9	NE	67.54	34.31	NE	NE	35.21	17.07
*Methicillin-Resistant S. aureus—MRSA*	4.22	15.81	33.77	17.15	67.21	17.7	17.60	8.53
*K. pneumoniae (ATCC13883)*	16.9	31.63	33.77	17.15	67.21	35.4	17.60	17.07
*S. aureus (ATCC25923)*	16.9	15.81	33.77	17.15	67.21	17.7	17.60	8.53
*C. parapsilosis (ATCC22019)*	33.8	15.81	16.88	34.31	33.60	70.8	35.21	34.15

NE, MIC value not found.

For the clinical isolate KPC1410503, the Cu-1a peptide and the analog R10b were efficient at low concentrations, while the analogs R1b, R2b, and R8b presented MIC values at high concentrations. MIC was not found for the analogs R1a, R3b, and R6b against KPC1410503 at the tested concentrations. Regarding the ATCC13883 strain of *K. pneumoniae*, the Cu-1a peptide and the analogs R2b, R8b, and R10b showed better results, while for peptides R1a, R1b, R3b, and R6b, MIC was found at high concentrations.

In general, most peptides showed activity against both strains of *K. pneumoniae*. However, Cu-1a and analog R10b showed the best activity against both strains. It was also observed that a lower concentration of Cu-1a and its analogs is necessary to inhibit the microbial growth of the ATCC13883 strain compared to the multidrug-resistant clinical isolate KPC1410503 (Table 3).

The Cu-1a peptide and its analogs were able to inhibit the growth of the multidrug-resistant strains KPC1410503 and MRSA3730592, as well as inhibit the growth of the reference ATCC strains of *K. pneumoniae* and *S. aureus*. It is important to note that this is the first report on the antimicrobial effect of the Cu-1a peptide against strains of *K. pneumoniae* and MRSA.

It was also observed that all peptides tested had antimicrobial effects against *C. parapsilosis* ATCC22019 at different concentrations. Peptides R1a and R1b showed better antifungal activity, showing MIC values at low concentrations when compared to Cu-1a peptide and analogs R2b, R3b, R6b, R8b, and R10b (Table 3). These results emphasize the activity of the peptide against bacteria and fungi, in addition to its efficacy against resistant multidrug microorganisms.

### RAW 264.7 and Hfib cell viability and NO production

**Figure 2:**
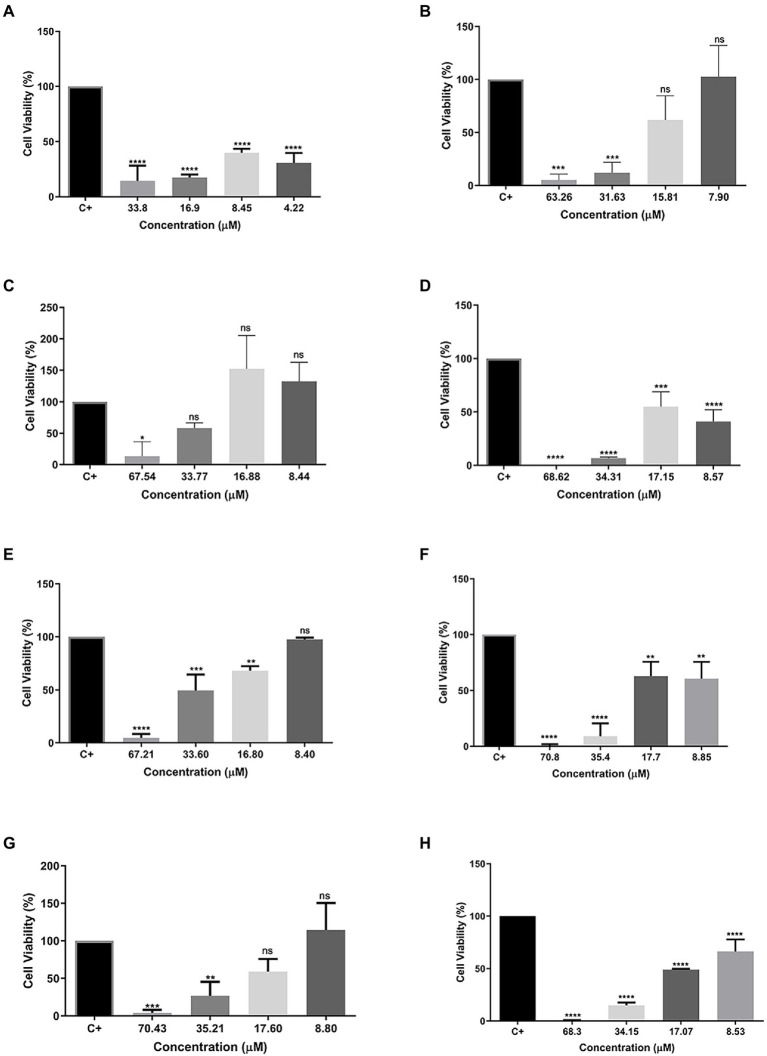
Percentage of cell viability in RAW 264.7 cells. **(A)** Cu-1a; **(B)** R1a; **(C)** R1b; **(D)** R2b; **(E)** R3b; **(F)** R6b; **(G)** R8b; and **(H)** R10b. One-way ANOVA—Bonferroni’s multiple comparisons test. Each bar represents the mean ± SD of cellular absorbance; *N* = 3. ^*^p < 0.05, ^**^p < 0.01, ^***^p < 0.001, ^****^p < 0.0001, and NS *p* > 0.05.

To evaluate the cytotoxicity of all tested peptides, the MTT assay was performed to evaluate cell viability. As expected, the peptide Cu-1a exhibited high toxicity at all tested concentrations (Figure 2A) against RAW264.7 cells. The analogous peptides showed different levels of toxicity against RAW264.7 cells (Figures 2B–H). Changes made in the sequence and size of the Cu-1a peptide were not effective in decreasing the cytotoxicity toward RAW264.7 cells in most of the designed peptides. Analogs R2b, R3b, R6b, and R10b (Figures 2C–H) showed high toxicity against macrophages at concentrations that were effective for the microorganisms evaluated, eliminating these as promising peptides for therapeutic agents like the original peptide. On the other hand, analogs R1a, R1b, and R8B were not toxic to RAW264.7 cells at the tested concentration. These results indicate that R1a, R1b, and R8b analog peptides can be further explored as promising therapeutic agents. Lastly, after 24 h, the NO production was analyzed in cell culture supernatant, and no NO production was observed in the presence of the peptides at all tested concentrations.

**Figure 3:**
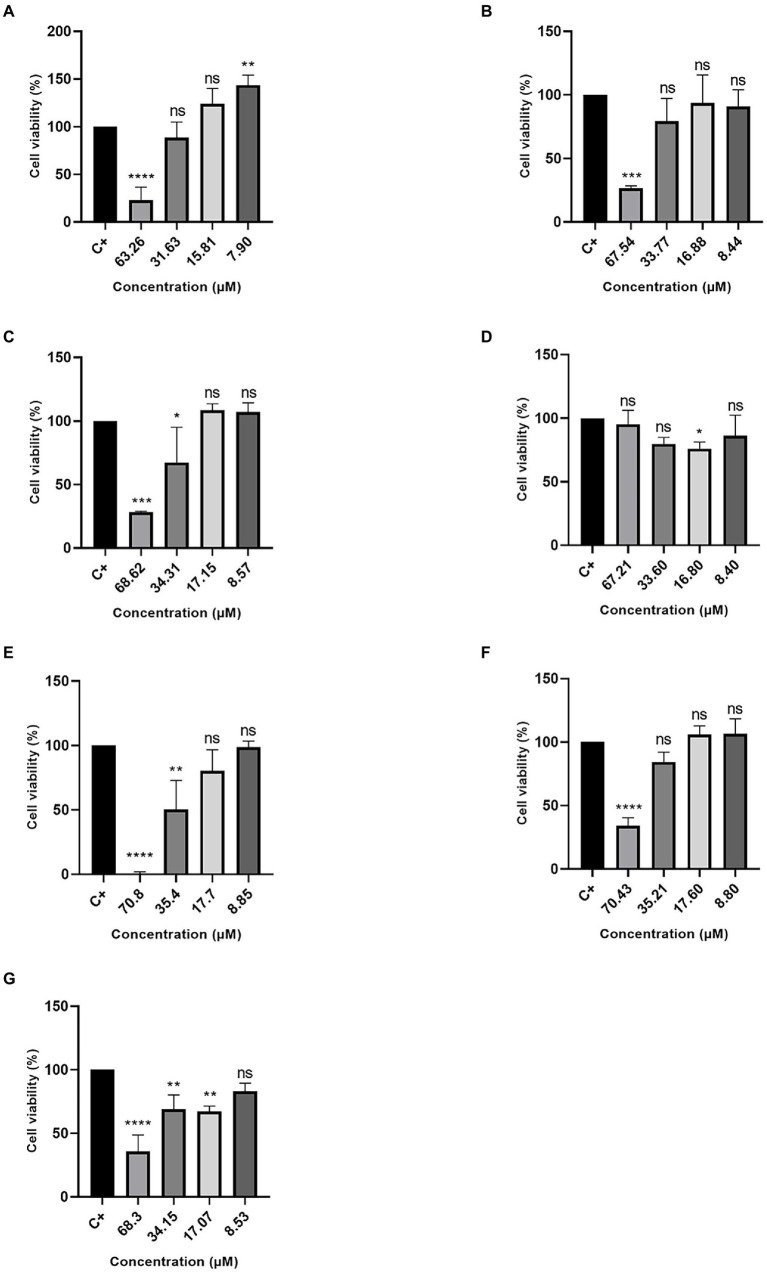
Percentage of Cell Viability in HFib cells. **(A)** R1a; **(B)** R1b; **(C)** R2b; **(D)** R3b; **(E)** R6b; **(F)** R8b; and **(G)** R10b. One-way ANOVA—Bonferroni’s multiple comparisons test. Each bar represents the mean ± SD of cellular absorbance; *N* = 3. ^*^p < 0.05, ^**^p < 0.01, ^***^p < 0.001, ^****^p < 0.0001, and NS *p* > 0.05.

Excluding the R3b peptide, all-analog peptides showed cytotoxicity against human fibroblast cells at the highest concentration (Figure 3). In addition to eradicating 100% of the cells at 70.8 μM, the R6b peptides also showed a slight activity at 35.4 μM, reducing the cell viability (Figure 3E). Although the R8b (Figure 3F) and R10b (Figure 3G) peptide analogs were toxic to Hfib cells, killing 100% of them at the highest concentration (70.43 and 68.3, respectively), they were not harmful to these cells at the other concentrations. However, these peptides remain to kill MCF-7 cell lines at the lowest concentrations. Regarding the tests performed with the MDA-MB-231 strain, the R1a analog peptide demonstrated activity against mammary adenocarcinoma cells (at 63.26, 31.63, and 15.81 μM), but R1a did not present a cytotoxic effect, at these concentrations, against Hfib cells, being toxic only at the highest concentration (63.26 μM). The R3b peptide analog was the only one, among all analogs, that did not show any cytotoxic effect on Hfib cells. Furthermore, this peptide showed activity against both MCF-7 and MDA-MB-231 (at the highest concentration tested). The data suggest that these analog peptides can be explored as a potential cancer-fighting molecule in the future.

### Cytotoxicity tests against cancer cell lines

**Figure 4:**
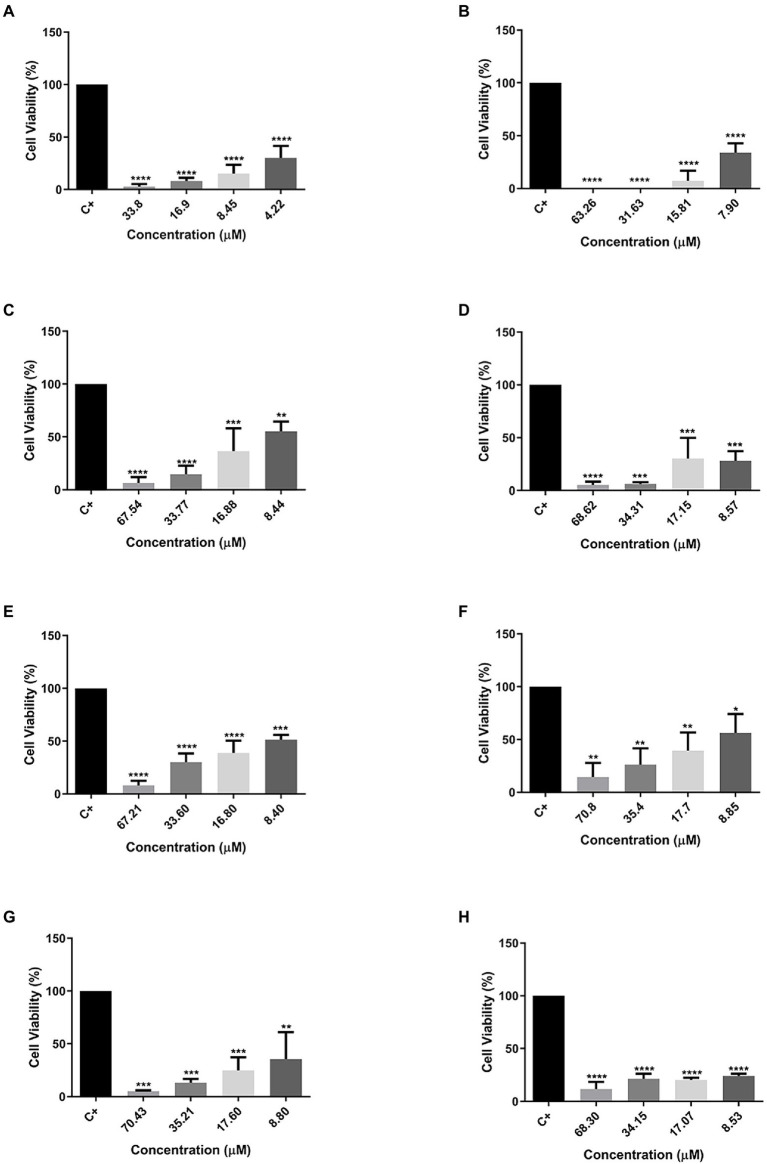
Percentage of cell viability in MCF-7 cancer cells. **(A)** Cu-1a; **(B)** R1a; **(C)** R1b; **(D)** R2b; **(E)** R3b; **(F)** R6b; **(G)** R8b; and **(H)** R10b. One-way ANOVA—Bonferroni’s multiple comparisons test. Each bar represents the mean ± SD of cellular absorbance; *N* = 3. ^*^p < 0.05, ^**^p < 0.01, ^***^p < 0.001, ^****^p < 0.0001, and NS *p* > 0.05.

**Figure 5:**
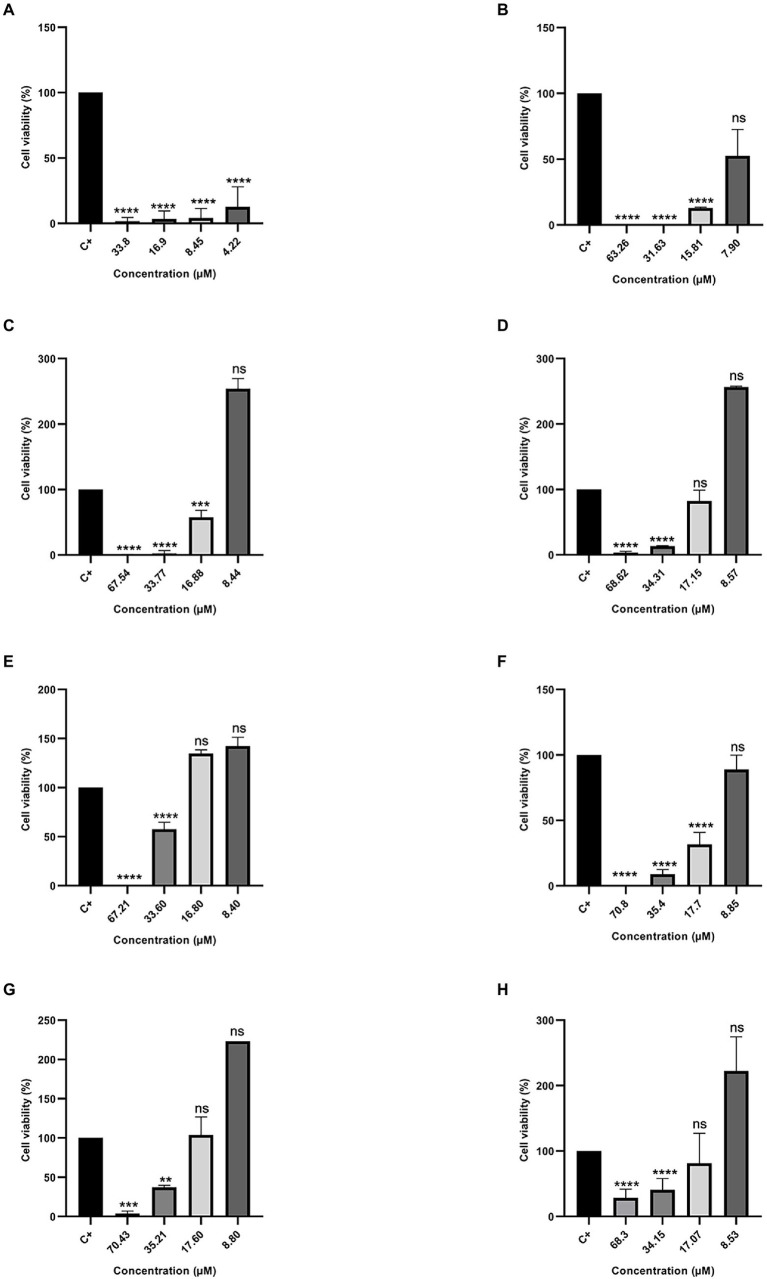
Percentage of Cell Viability in MDA-MB231 cancer cells. **(A)** Cu-1a; **(B)** R1a; **(C)** R1b; **(D)** R2b; **(E)** R3b; **(F)** R6b; **(G)** R8b; and **(H)** R10b. One-way ANOVA—Bonferroni’s multiple comparisons test. Each bar represents the mean ± SD of cellular absorbance; *N* = 3. ^*^p < 0.05, ^**^p < 0.01, ^***^p < 0.001, ^****^p < 0.0001, and NS *p* > 0.05.

The MTT assay was also used to evaluate the cytotoxic effect of peptides against two cellular types of mammary adenocarcinoma, and MCF-7 was chosen as the standard cell (Figure 4) and MDA-MB231 (Figure 5) as a cell resistant to antitumor agents. The peptide Cu-1a showed activity against both tumor cells (Figures 4A, 5A), even at low concentrations. In addition, all analogs demonstrated antitumor potential against MCF-7 in all tested concentrations (Figures 4B–H). They were highly effective, as it is possible to observe cell viability below 50% even at lower concentrations.

On the other hand, different results were found when peptides were tested against the therapeutic agent resistant to antitumor MDAMB-231 (Figure 5). The peptide Cu-1a continued to show activity against the therapeutic agent-resistant breast cancer cell line (MDAMB-231), even at low concentrations, while the analog peptides demonstrated antitumor activity against this line only at higher concentrations. The data obtained with the two tumor strains show that the analog peptides did not lose their antitumor potential after the removal and replacement of amino acid residues from the original sequence, but they were not able to perform as well as peptide Cu-1a against resistant tumor cells.

### *In vitro* test against insect cells

The insecticidal activity was tested against line cells of the corn caterpillar *Spodoptera frugiperda* (SF21), an important agricultural pest capable of producing damage in several commercial crops worldwide.

**Figure 6:**
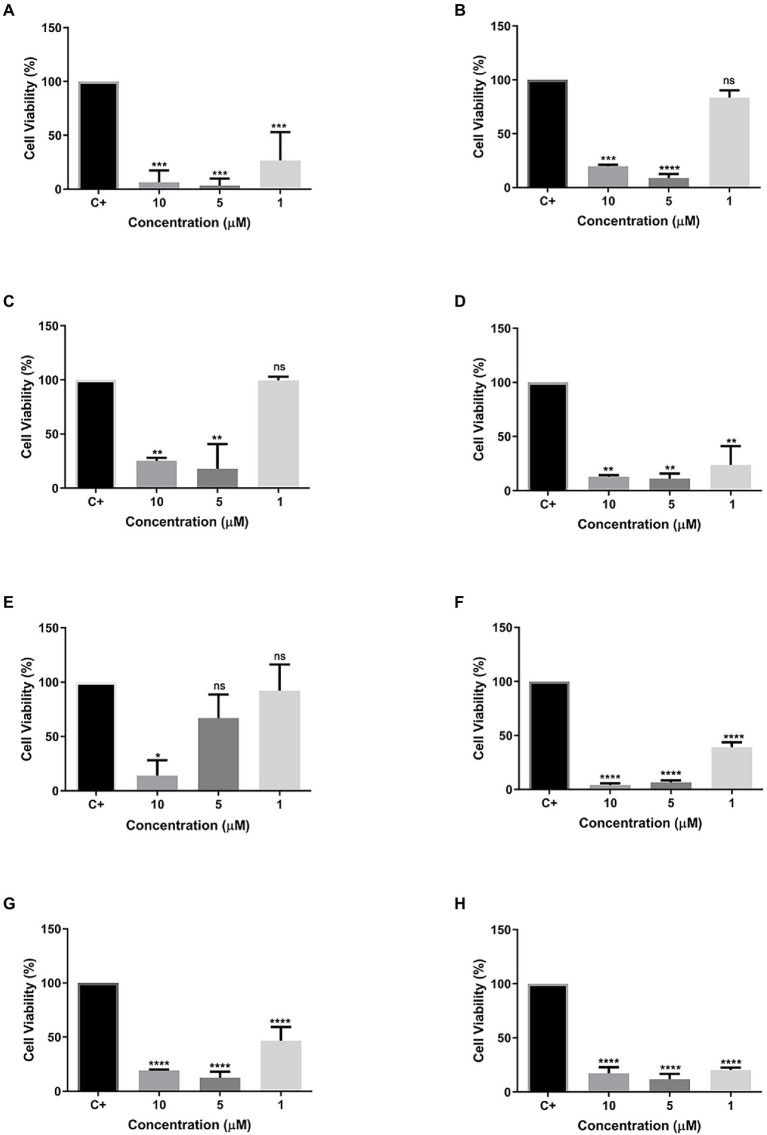
Cell viability of synthetic peptides tested against SF21 cell line at concentrations of 10, 5, and 1 μM. **(A)** Cu-1a; **(B)** R1a; **(C)** R1b; **(D)** R2b; **(E)** R3b; **(F)** R6b; **(G)** R8b; and **(H)** R10b. One-way ANOVA—Bonferroni’s multiple comparisons test. Each bar represents the mean ± SD of cellular absorbance; *N* = 3. ^*^p < 0.05, ^**^p < 0.01, ^***^p < 0.001, ^****^p < 0.0001, and NS *p* > 0.05.

Different concentrations (10, 5, and 1 μM) of all eight peptides were used. Cells were then evaluated for their cell viability after 24 h of contact with the different peptide concentrations. Most of the tested peptides showed cytotoxic activity at all concentrations, except for peptides R1a, R1b, and R3b (Figures 6B–E), which presented low toxicity. The peptide Cu-1a (Figure 6A) and the analogs R2b, R6b, R8b, and R10b (Figures 6D,F–H) were toxic to insect cells in all tested concentrations but were more efficient at higher concentrations. Moreover, peptide R3b (Figure 6E) showed lower efficiency (14% viable cells) only at a concentration of 10 μM. At concentrations of 5 and 1 μM, cell viability was greater than 50%. These results demonstrate the insecticide potential for the peptide Cu-1a and its analogs R2b, R6b, R8b, and R10b, which can be promising for further studies on their insecticidal activity.

## Discussion

### Rational design of peptides and helical wheel peptide projection

The rational design of peptides consists of several physicochemical parameters previously analyzed for projection and creation of new peptides with antimicrobial activities and reduction of unwanted activities, such as hemolysis or toxicity toward mammalian cells (Ya’u and Nujarin, 2020). Currently, there are three main approaches mostly used for the projection of new peptides: based on templates, physicochemical properties, and *de novo*. Within these approaches, important structural characteristics for antimicrobial peptide activity are analyzed, such as size, hydrophobicity, conformation, charge, and amino acid sequence (Fjell et al., 2012).

The objective of this research was to perform rational design of molecules from a protein with potential for application in different biotechnological areas. For this, we chose the peptide Cu-1a described by Kuhn-Nentwig et al. (2002) because it adapted to all the parameters, we sought for the model molecule. We observed that this peptide has biotechnological potential, but has high toxicity, which causes this peptide to be discarded due to applicability issues. We thus designed seven analogous molecules, aiming to reduce the cytotoxic effect without losing the biological activities described in the literature. Changes were performed in sequence size and amino acid residues, valuing the hydrophobicity, helicity, cationicity, and reduction of cytotoxic activity in analog peptides, since these parameters are important for Cu-1a activity.

For a better visualization of the modifications made in the analog peptides, we chose helical wheels (Figure 1) because they are a type of plot widely used to visualize the structural conformations adopted by peptides. In this type of plot, it is possible to observe secondary structures of the alpha-helix type adopted by some peptides. In addition, this allows the researcher to have a panoramic view of helical peptides, with the amino acid residues interacting with each other and adopting the shape of a perfect circle, which allows the visualization of the hydrophobic and hydrophilic faces of the molecules (Schiffer and Edmundson, 1967; Wadhwa et al., 2018).

Through helical wheels, we can perceive all analogs with a larger hydrophobic face which can contribute to interactions of the molecule with different cell types. Peptide hydrophobicity influences the activity and selectivity of AMPs. Increased hydrophobicity can increase antimicrobial activity, and its reduction leads to decreased antimicrobial effect. In addition, hydrophobicity can lead to a variety of target cells from a peptide (Almsned, 2017). Thus, hydrophobicity and charge were factors considered for the design of synthetic analog peptides.

### Antimicrobial activity against bacteria and fungi

Previous literature has described the antimicrobial activity of peptide Cu-1a against ATCC strains of *Escherichia coli, S. aureus, Enterococcus faecalis*, and *Pseudomonas aeruginosa* (Kuhn-Nentwig et al., 2002). However, there was no information regarding its antimicrobial activity against multidrug-resistant clinical isolates such as KPC and MRSA and against fungi. Similar antimicrobial effects against *K. pneumoniae* ATCC strains have been previously described by Kuhn-Nentwig et al. (2002). This peptide also presented an antimicrobial effect against clinical isolates of KPC and MRSA. Antimicrobial activity was observed against these strains and against the yeast *C. parapsilosis* by the analogs developed from peptides. The peptides that showed the best microbial activity were Cu-1a and analog R10b, which obtained MICs found in low concentrations for all tested organisms.

Antimicrobial tests demonstrated that changes in analog peptides when compared to Cu-1a resulted in reduced antimicrobial activity when tested against the clinical isolate of KPC1410503 at lower concentrations, and MIC was observed only at higher concentrations. Many multidrug-resistant strains of KPC have a thicker capsule than strains with little resistance, hindering the activity of antimicrobials that act on the membrane (Nepal et al., 2017). That was the case of the clinical isolate used in this study, but this was not observed for the analog R10b, where the substitution of Ala-4 by Lys-4, Phe-8 by Arg-8, Lys-12 by Ala-12, and Thr-16 by Ala-16 resulted in an increase in hydrophobicity and + 5 load. These modifications were important for the activity of this analog against this strain, and a MIC close to the concentration of the original peptide was found. Thus, we can conclude that the alterations made in the analog R10b contributed to the selectivity and activity of this peptide in relation to this multidrug-resistant strain of KPC1410503.

When tested against strain ATCC13883 of *K. pneumoniae* whose resistance profile is low and with the absence of a capsule, we observed that the peptides R2b and R8b showed better activity, with MIC at low concentrations, while the peptide Cu-1a and the analog R10b maintained their activities against this strain. Alterations realized in the analog R10b contributed to its activity and selectivity against multidrug-resistant strains of this bacterial species. It was observed that the alteration of Thr-16 by Ala-16 in the analogs R2b, R8b, and R10b resulted in an improvement in activity against *K. pneumoniae* ATCC13883. In addition, the substitution of Lys-12 by Ala-12 was important for R8b and R10b activity against this strain. The substitution of Ala-4 by Lys-4 was important to maintain the MIC against KPC1410503 in a non-resistant strain, ATCC13883, which was not observed for analogs R2b and R8b; these did not have this substitution and the MIC for KPC1410503 was only found in high concentrations.

There was no difference in MIC concentration found for the two strains of *S. aureus* when exposed to different concentrations of analog peptides; however, the MIC found for Cu-1a versus MRSA3730592 was better than that observed for the non-resistant strain, demonstrating that this peptide obtained greater selectivity against this strain. The analogs that showed the best activity against both microbial strains were R1a, R2b, R6b, R8b, and R10b, with the analog R10b showing better microbial activity against both strains.

The analog peptide R10 showed better activity against all bacterial strains tested, demonstrating that the alterations performed maintained its activity against gram-positive and gram-negative bacteria, making it the most promising among the analog peptides against bacteria. Lys and Arg are the preferred amino acids during the rational design of molecules with more efficient antimicrobial actions, but the presence of Arg residues is accompanied by high hemolytic properties when compared to Lys-rich peptides (Wiradharma et al., 2011; Ong et al., 2013). We performed the interleaved use of Lys and Arg additions following the analog peptide R10b, which contributed to selectivity against microbial agents, and when tested in RAW cells, it was observed that at the MIC value found for bacteria, there is low toxicity in RAW cells, with more than 50% cell viability at concentrations of 17.07 and 8.53 μM.

When tested against fungi, the analog peptides that stood out were R1a and R1b with MIC at low concentrations, demonstrating that the alterations made in these peptides favored their selectivity against fungal strains. This was not found for the original peptide, which had activity at higher concentrations. In these two peptides, the alterations were only in the last amino acid residue, where in R1a Lys-19, it was maintained and in R1b this residue was removed, but this alteration did not result in loss of activity against fungi, demonstrating that the structural arrangement resulted in greater selectivity against fungi. Substitutions of amino acid residues along the sequence in other analog peptides resulted in the loss of antifungal activity, and activity was observed only at high concentrations.

In a study conducted by Wiradharma et al. (2011), with α-helical AMPs of unnatural origin, these peptides were cationic and amphiphilic and were designed to mimic the behavior of α-helical AMPs of natural occurrence. In this study, the peptide LLKK3 caught our attention because it presented the best activity against *C. albicans*. This peptide is rich in residues of Leu and Lys, and our analogs R1a and R1b have these residues arranged in its sequence. When comparing our data, we observed that, just like the peptide LLKK3, our analogs R1a and R1b have an appropriate hydrophobic-cationic balance to form an α-helical conformation in a membrane-like environment besides having an ideal amphiphilic helical conformation that can be seen in the helical wheel projection. Therefore, it is suggested that these residues collaborate with the structural arrangement adopted by these peptides in fungal cells. Despite maintaining the hydrophobic-cationic balance in the other analog peptides when performing changes in amino acid residues, the structural arrangement undergoes alteration, and this interferes with the interaction of peptides with fungal cells, leading to decreased activity.

### Cytotoxicity RAW cells compared to other cell types

To verify the toxic effect of the analog peptides after the modifications, we performed the cell cytotoxicity assay in RAW264.7 cells and observed that the peptide Cu-1a was toxic in all tested concentrations, confirming the cytotoxic effect of this molecule. The first 19 residues from the primary sequence of Cu-1a form the initial part of the toxic N-terminal of Cu-1a (Kuhn-Nentwig et al., 2013), and these were the basis for the generation of the R1a analog. From this, the modifications were made along the sequence for the generation of other analogues, in view of the reduction of toxicity already reported previously in the literature.

The modifications made to the peptide sequences resulted in a reduction in the toxicity observed on RAW264.7 macrophages at lower concentrations. When we removed Lys-19 from the sequence to generate the analog R1b, we observed a reduction in toxicity from 33.77 μM, with 58% of viable cells, and this is not observed in the R1a analog, which presents high toxicity in this concentration range. Substitutions of amino acid residues along the peptide chain reduced the cellular toxicity observed for the analog peptides R2b, R3b, R6b, R8b, and R10b at lower concentrations, and it is possible to visualize cell viability equal to or greater than 50%.

The results of cytotoxic tests demonstrate that the analog R1a could be used in the control of MRSA, ATCC25923 of *S. aureus,* and ATCC22019 of *C. parapsilosis*, because we observed 63% of viable cells in the MIC found for these strains. R1b could be used to control all the microorganisms tested, except for KPC1410503, where high toxicity of this peptide is observed in the MIC concentration found for this strain. For the analog R2b, cell viability was observed above 50% in the MIC found for MRSA3730592, ATCC25923 of *S. aureus*, ATCC13883 of *K. pneumoniae*, and can be used to control these pathogens.

R3b was the only analog that presented MIC at high concentrations against all tested microorganisms, being toxic to RAW264.7 cells in this concentration. R6b could be used only to control the two strains of *S. aureus* tested, and cell viability of 62% was observed in the MIC found for these two strains. From the MIC concentration found for MRSA3730592, ATCC25923 of *S. aureus,* and ATCC13883 of *K. pneumoniae*, cell viability of 59% was observed in RAW264.7 cells when exposed to the analog R8b.

Still analyzing Figure 2 in comparison with Table 3, the analog R10b showed better antimicrobial activity against all bacteria tested when compared to other analog peptides. It was demonstrated that the substitutions performed in the sequence contributed to a greater selectivity of the peptide against bacteria and can be used in the control of these bacteria, since cell viability is observed above 50% in RAW264 cells. This analog also stood out when tested against MCF-7 and Insect Cells SF-21, demonstrating it to be a peptide with various biotechnological applications.

### Cytotoxicity Hfib cells

Hfib cells were used to assess the cytotoxic potential of analog peptides (Figure 3). The data showed that, with the exception of the R3b analog (Figure 3D), the remaining peptides were toxic, at some level, to cells when tested with the highest concentration of each analog peptide. However, in the other concentrations tested in this work, the analogs did not show considerable cytotoxicity. Previous tests have shown that cupiennin 1a has hemolytic activity against human erythrocytes at a semi-maximal concentration (EC_50_) of 24.4 μM (Kuhn-Nentwig et al., 2002). Tests performed against mouse skeletal myoblasts (L-6 cells) demonstrated that the peptide cupiennin 1a is toxic to these cells at a concentration of 0.342 uM (EC_50_; Kuhn-Nentwig et al., 2011). The concentrations tested in this work are higher than those used in these studies. Thus, it is possible to suggest that cupiennin 1a analog peptides maintain the potential anticancer activity, without being toxic to healthy cells, even at higher concentrations.

### Cytotoxicity tests against cancer cell lines

Kuhn-Nentwig et al. (2011) published a study in which they demonstrated that Cu-1a has activity against different human leukemic cell lines, as well as being able to eradicate HeLa cells, both in *in vitro* tests. In this work, we tested the antitumor activity of Cu-1a and its analogs through MTT assays and evaluated cell viability after exposure to different peptide concentrations against two cell lines of breast cancer. MCF-7 was chosen as a strain non-resistant to therapeutic agents, and MDAMB-231 was chosen as resistant strain, to evaluate peptide behavior when exposed to two different cancer strains. We expected to find activity of the peptide Cu-1a and its analogs against cell lines of breast adenocarcinoma. In this work, we were able to confirm the activity against tumor cells for the peptide Cu-1a and its analogs.

The analog peptides that showed the best activity against MCF-7 were R1a and R10b, and it was possible to observe low cell viability in all concentrations tested. On the other hand, only the analog R1a maintained activity when tested with the tumor cell MDAMB-231, a breast cancer cell resistant to therapeutic agents. Although the resistance factor influences the action of AMPs on cell growth, it is necessary to consider the sequence of peptides and how their structural arrangement occurs in cell membranes. In this context, when analyzing the changes made along the R10b peptide sequence with the observed cytotoxicity, it is seen that these changes led to the loss of the activity of this peptide in more resistant tumor cells, which was not observed for the analog R1a that did not undergo such changes in its sequence and maintained its activity.

Although toxicity to MCF-7 is observed for the other analog peptides, the results indicate that they do not have selectivity for resistant tumor cells, as with the R10b analog mentioned above. So, we can infer that changes in amino acid residues along the chain of other analog peptides also resulted in the loss of their activity against the resistant lineage MDAMB-231, where the other analog peptides were toxic only at the highest concentrations. The secondary structure of projected peptides affects the potency, selectivity, and structural orientation of the peptides, depending on the orientation angle, while the arrangement adopted by the peptide produces destabilization of the membrane phospholipids and affects permeability (Hanaoka et al., 2016; Liscano et al., 2020). Therefore, very large changes along the peptide chain can influence these interactions and consequently lead to loss of activity, depending on the cell type to which it is exposed.

### *In vitro* test against insect cells

The insecticidal activity of Cu-1a was demonstrated by injecting the peptide in *Drosophila melanogaster*. Although the methodology and the organism used to assess insecticidal activity are different, we can see that our data corroborate those of Kuhn-Nentwig et al. (2002) on the insecticidal activity shown by the peptide Cu-1a. The authors report that it took 5.9 pmol of peptide per mg of fly to obtain the EC50.

We performed cell viability tests to evaluate the insecticidal effect of Cu-1a and its analogs against the cell line of *Spodoptera frugiperda* (SF-21). The peptide Cu-1a showed high cellular toxicity against the SF-21 strain in all tested concentrations (Figure 5A), thus confirming its insecticidal potential, as described by Kuhn-Nentwig et al. (2002).

The analog peptides showed cellular toxicity against SF-21 (Figures 6B–D,F). The analogs R1a and R1b showed toxicity only when tested at concentrations of 10 and 5 μM and were not toxic with 1 μM peptide. The analog R3b was toxic to cells only at the highest concentration tested. The peptides R2b, R6b, R8b, and R10 had a higher cytotoxic effect on insect cells, and it was possible to observe cell viability below 50% in the lowest concentration tested (1 μM). However, using the methodology used here, *in vivo* tests will be necessary to confirm the results presented here as insecticidal activity of these molecules. However, the results obtained in the cell viability assay give us an indication that these peptides have insecticidal potential.

## Conclusion

The analogs that presented the best biological activities against different cell types and that presented the least toxic effect were R8b and R10b. These peptides stand out from the others because, in addition to having activity against bacteria and fungi, they also presented activity against tumor cells MCF-7 and insect cells SF-21 in all concentrations tested. In addition, they showed low toxicity in RAW264.7 and Hfib cells at different concentrations tested. Thus, we can conclude that substitutions performed on these peptides were able to maintain the broad spectrum of biological activity previously reported for Cu-1a, besides having contributed to a reduction in the toxicity. Rational design from potential molecules of natural origin contributes strongly to the development of new molecules with different biotechnological applications.

## Data availability statement

The datasets presented in this study can be found in online repositories. The names of the repository/repositories and accession number(s) can be found at: https://page.ucb.br/bc/pesquisador.listaProducoes?idc=39177&id1=106&id2=2018, UCB 34322.

## Author contributions

RA, MR, and SD: conceptualization. RA, TD, and ML: methodology. RA, MR, NB, TR, and SD: validation. SD: resources. RA, TR, ML, and NB: data curation. RA: writing—original draft preparation. RA, TR, NB, MR, and SD: writing—reviewing and editing. SD and MR: supervision. All authors contributed to the article and approved the submitted version.

## Funding

This work was supported by Coordenação de Aperfeiçoamento de Pessoal de Nível Superior (CAPES).

## Conflict of interest

The authors declare that the research was conducted in the absence of any commercial or financial relationships that could be construed as a potential conflict of interest.

## Publisher’s note

All claims expressed in this article are solely those of the authors and do not necessarily represent those of their affiliated organizations, or those of the publisher, the editors and the reviewers. Any product that may be evaluated in this article, or claim that may be made by its manufacturer, is not guaranteed or endorsed by the publisher.

## Footnotes

^1^ http://lbqp.unb.br/NetWheels/

^2^ http://lbqp.unb.br/NetWheels/
